# Navigational Transmaxillary Endoscopic Approach for Inferomedial Tumors

**DOI:** 10.3389/fonc.2022.804070

**Published:** 2022-04-27

**Authors:** Cheng-Hsien Wu, Yi-Yun Ho, Tzu-Lun Liu, Tzu-Ying Wu, Han-Chieh Cheng, Chieh-Chih Tsai

**Affiliations:** ^1^ Oral and Maxillofacial Surgery, Department of Stomatology, Taipei Veterans General Hospital, Taipei, Taiwan; ^2^ Department of Dentistry, School of Dentistry, National Yang Ming Chiao Tung University, Taipei, Taiwan; ^3^ Orthodontic and Pediatric Dentistry, Department of Dentistry, Taipei Veterans General Hospital, Taipei, Taiwan; ^4^ Department of Ophthalmology, Taipei Veterans General Hospital, Taipei, Taiwan; ^5^ Department of Ophthalmology, School of Medicine, National Yang-Ming Chiao Tung University, Taipei, Taiwan

**Keywords:** transmaxillary, endoscope, orbital tumor, navigation, computer assisted, minimally invasive

## Abstract

Orbital tumors encompass a heterogeneous range of histopathology and usually variable in location. Traditionally, transconjunctival medial orbitotomy is used to access the medial orbital wall. However, it creates potential risk of soft tissue sequelae such as scarring, lid contracture, or entropion/ectropion. For the lesions close to the orbital apex, increased risk of optical nerve injury should be cautious during orbitotomy procedure. Transnasal endoscopic approach to the orbital walls has been applied since 1999. Although it provides good surgical visualization and prevents the soft tissue and neural complications, the narrow nasal corridor increases the surgical complexity. Extensive sphenoethmoidectomy is usually required to gaining access. Furthermore, the resultant medical orbital defect is difficult to repair. The maxillary sinus is the largest paranasal sinuses which is located beneath the orbital floor. It provides an ample working space for instrumentation. Meanwhile, repair of the orbital floor defect is feasible and with high degree of accuracy under navigation control. In this report, we propose a novel computer-assisted endoscopic protocol to excise the medial orbital tumors with immediate repair of the wall defect.

## 1 Introduction

Orbital tumors encompass a heterogeneous range of histopathology, which 64% are benign and 46% are malignant according to a large cohort survey (1). According to its relationship with the extraocular muscle cones, the orbital tumors are classically divided into intraconal and extraconal position (2). Surgical excision is sometimes challenging to achieve balance among the optimal operative visualization, preserving function and minimize morbidities. Several transorbital and extraorbital surgical approaches has been developed for orbital tumor excision (3). However, for deep-seated lesions or those close to the orbital apex, the surgical filed is usually confined and risky to the surrounding neurovascular structures. Traditionally, surgical approaches to the lesions located in the medioposterior orbit are transconjunctival medial orbitotomy, and medial lateral orbitotomy (3–5). Although those approaches are straightforward, however, the soft tissue sequalae secondary to scarring e.g. lid retraction, ectropion/entropion, epiphora may anticipate (6). For large medial orbital lesions or those located close to the orbital apex, increased risk of optic nerve compressive injury during dissection should not be overlooked. Since the rapid progress of sinonasal endoscopic surgery, this technique is gradually applied in the orbital tumor or infectious condition management (7). The paranasal sinuses located at the medial and inferior parts of the orbital cavity provide a natural surgical access to the orbital tumors. The first case of transnasal endoscopic approach (TNEA) excision of the medioposterior orbital cavernous hemangioma was reported in 1999 (8). Although the nasal cavity provides a natural corridor for surgical access to the medial orbital wall, extensive sphenoethmoidectomy is usually required to gain better visualization and surgical freedom (9). In some cases, a second surgeon is needed for additional suction and irrigation procedures to improve visualization. This may require a septoplasty for additional instrumentation and the four-hands technique further increase the complexity of the operation (10). The maxillary sinus is the largest paranasal sinuses and located just beneath the orbital floor. Naturally, it provides a good access to the orbital floor and even to the medial and lateral walls. By using the traditional Caldwell Luc’s approach, this wide opening sinus window become the direct portal to the orbital floor. In this report, we demonstrated the algorithm of the computer-assisted transmaxillary endoscopic approach (TMEA). The technical part will be explicated in four orbital tumor cases.

## 2 Materials and Methods

Four cases with tumors located in the inferomedial compartment of orbit were enrolled. Written informed consent was obtained from these patients for the publication of any potentially identifiable images or data included in this article. The demographic data and presenting symptoms/signs were listed in the **Table 1**. The image-guided transmaxillary surgical scheme is proposed in the following sequence: image processing and virtual model creation, virtual surgical simulation and transfer, navigational TMEA, and post-operative evaluation. The step-by-step workflow is listed below:

**Table 1:** Clinical characteristics and surgical parameters.

	Patient No. 1	Patient No. 2	Patient No. 3	Patient No. 4	
**Clinical parameters**					Average
Age (years)	85	64	48	51	62 ± 14.6
Gender	Male	Female	Female	Male	
Localization	Intraconal	Extraconal	Extraconal	Intraconal	
Laterality	Right	Left	Left	Right	
Quadrant of the orbit	Inferomedial	Inferomedial	Inferomedial	Superomedial/inferomedial	
Symptoms & signs	Near complete vision loss, proptosis	Progressive blurred vision and mild proptosis	Painful swelling	Progressive blurred vision and proptosis	
Histopathology	Schwannoma	Cavernous venous malformation	Idiopathic orbital inflammation	Cavernous venous malformation	
Size (cm^3^)	13.4 (2.8x2.0x2.4)	1.2 (1.3x1.0x0.9)	2.3 (1.2x0.9x2.1)	7.8 (2.0x2.3x1.7)	6.2 ± 4.9
Hospital stays (days)	4	3	3	5	3.8 ± 0.8
**Surgical parameters**					
Operation time (min)	155	180	174	300	202.3 ± 57.2
Blood loss (ml)	65	20	30	170	71.3 ± 59.4
Orbital floor reconstruction	Bio-membrane	Bio-membrane with titanium mesh	None	MEDPORE	
Early complication	Paranasal paresthesia (+) for 2 months with complete recovery	Paranasal paresthesia (+) for 1 month with complete recovery	Paranasal paresthesia (+) for 1 month with complete recovery	Paranasal paresthesia (+) for at least 2 months with progressive recovery	
	Nasal congestion (+) 3 weeks	Nasal congestion (-)	Nasal congestion (+) for 1month	Nasal congestion (+) for 1month	
	Diplopia cannot evaluate due to poor vision	Diplopia (+), complete resolve within 2 months	Diplopia (+), complete resolve within 2 months	Diplopia (+) for at least 2 months with progressive recovery	
Late complications	Mild enophthalmos	None	None	Mild enophthalmos	

### 2.1 Image Processing and Virtual Model Creation

**Figure 1:**
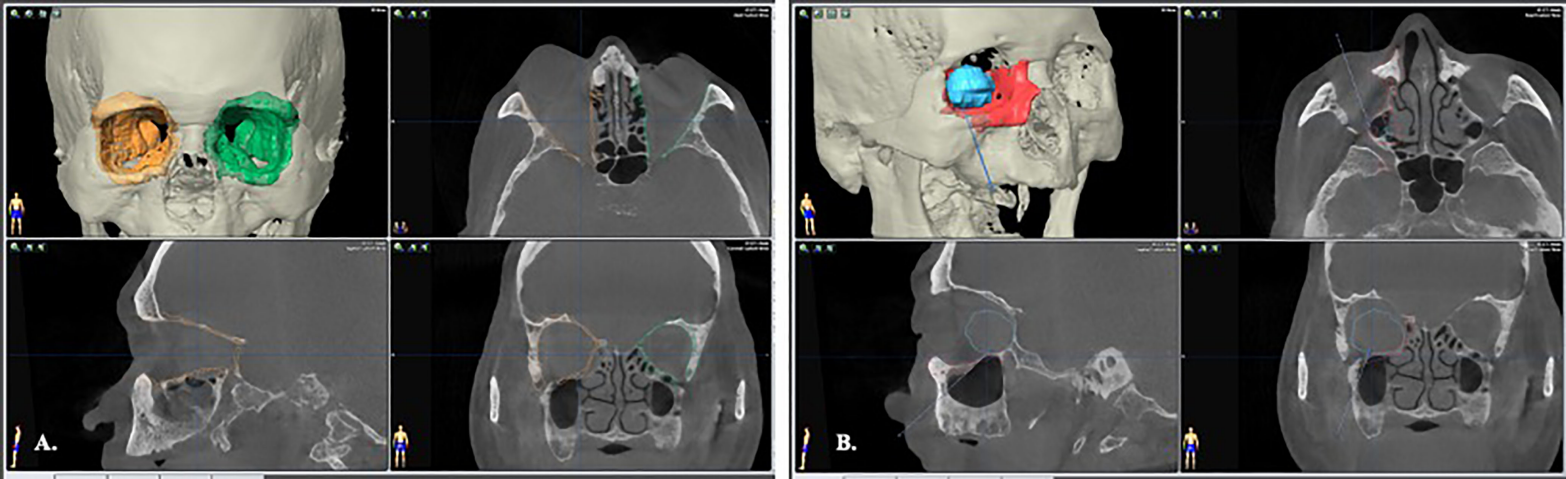
Image segmentation by bone-specific Hounsfield units. **(A)** segmentation of the normal, non-destructive orbital wall (green); the mirrored image from the contralateral normal orbital wall to rebuild the destructed right side medio-inferior orbital walls (orange). **(B)** The orbital tumor was segmented with fusion MRI and CT images to optimize the tumor boundaries (blue). A trajectory through the maxillary sinus that indicated the path of entry to orbital tumor was designed for intraoperative guidance.

Creation of an ideal virtual model is all the beginning of computer-assisted surgery. To optimize the skeletal image rendering quality, the volumetric CBCT scan with slice thickness of 0.3mm were used (NewTom NT, QR, Verona, Italy). The orbital tumors were best visualized with MRI sequences as the T1W, T1W with contrast and fat suppression technique, and T2W. The digital imaging and communications in medicine (DICOM) data were imported to the iPlan^®^ CMF Version 3.0 software platform (Brainlab AG, Feldkirchen, Germany) for subsequent planning. Automatic image fusion algorithm is ran first to coordinate the image datasets (11). This is a critical step that ensure all the objects created from different image datasets will register in the same spatial cranial position. Segmentation of the craniofacial bones was then done by the preset templates of the software or manual segmentation of the region of interest by bone-specific Hounsfield units (**Figure 1A**, green). The tumor was mapped based on the MRI for better soft tissue and tumor border delineation. If the orbital walls surrounding the tumor are destructed, a mirrored image is created from the healthy orbit and superimposed onto the affected side (**Figure 1A**, orange). Then surgeon could fine-tune the position of the mirrored object and outlines the margins to achieve symmetry. This will facilitate intraoperative guidance by printing out the physical model for pre-contouring the reconstructive material, plus by real-time navigation to ensure the implant position. The trajectories design allows surgeon to design the path of entry to the medial orbit and define the ideal entry point on the sinus roof/orbital floor (**Figure 1B**). The virtual surgical plan is then transferred to the navigator for intraoperative guidance. The according 3D segmented objects are exported in the form of stereolithographic (STL) files to manufacturing the physical models.

### 2.2 Navigational Transmaxillary Endoscopic Approach

#### 2.2.1 Navigation Coordinate Registration

The VSP done by the iPlan was imported into the infrared-based navigation system (Kick^®^, Brainlab AG, Feldkirchen, Germany). This stereoscopic infrared camera provides real-time tracking and displaying instrument positions intraoperatively. To ensure the operation is in the same coordinate system of the navigation image datasets, registration is the crucial preliminary step to re-orient the patient in the operating theatre. A “skull reference frame” with reflective marker spheres is fixed onto the patient’s skull. Then the registration is completed by using the optical surface matching device (Z touch^®^, Brainlab AG, Feldkirchen, Germany) plotting the skin points on the T zone of the forehead and nasal region. Optical tracking pointer is used to verify the accuracy of registration. The resulting point-to-point target registration error (TRE) should less than 1mm to optimize the accuracy of registration (12, 13).

#### 2.2.2 Caldwell Luc Approach and Trajectorial-Guided Orbital Floor Entry

**Figure 2:**
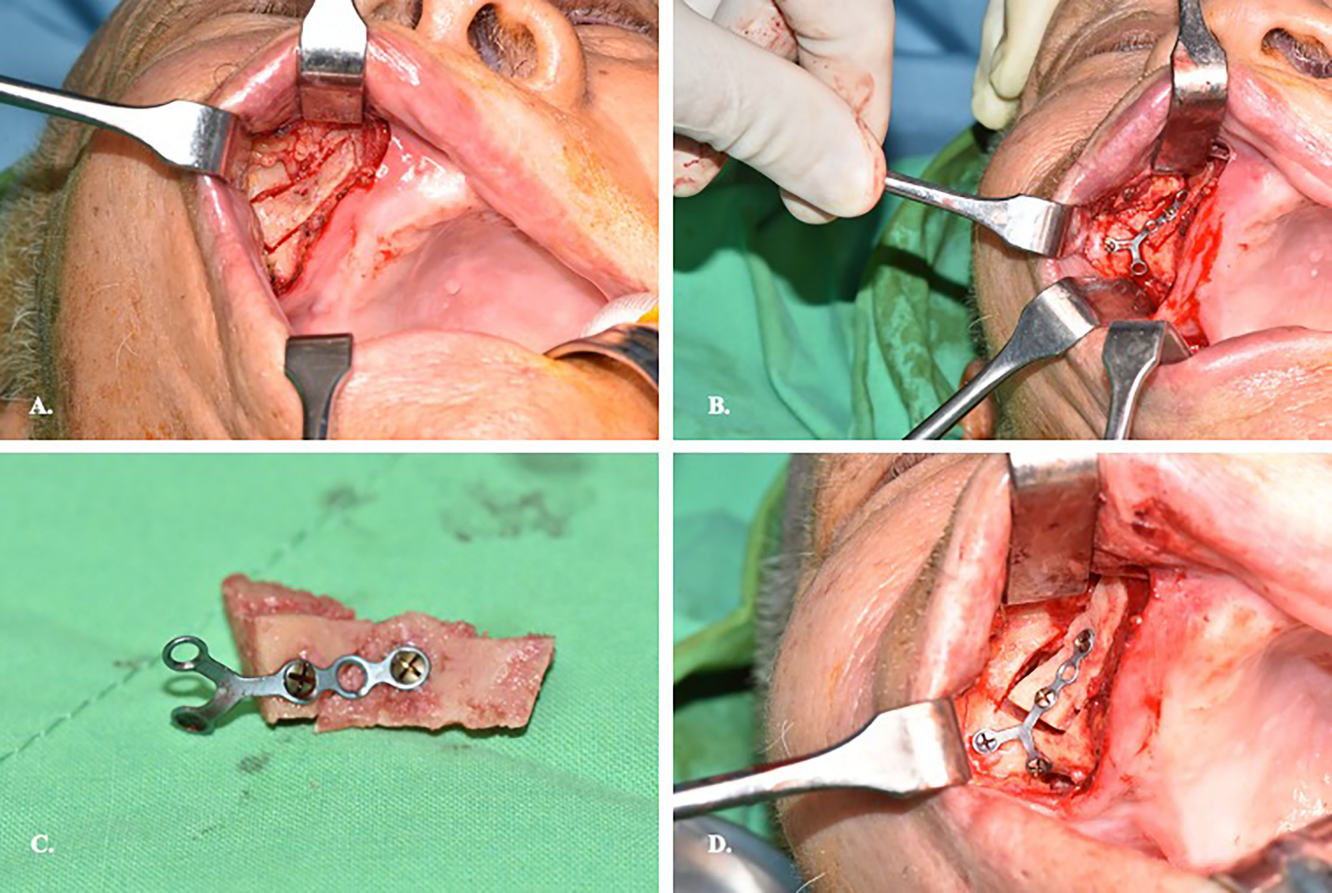
Preparation of the bone window on the anterior wall of maxillary sinus. **(A)** Supravestibular incision to expose sinus anterior wall. **(B)** Prebend the miniplate before osteotomy of the sinus wall. **(C)** Illustration of the miniplate-prefix bony wall. **(D)** Reposition of the bony wall after completion of the orbital surgery.

**Figure 3:**
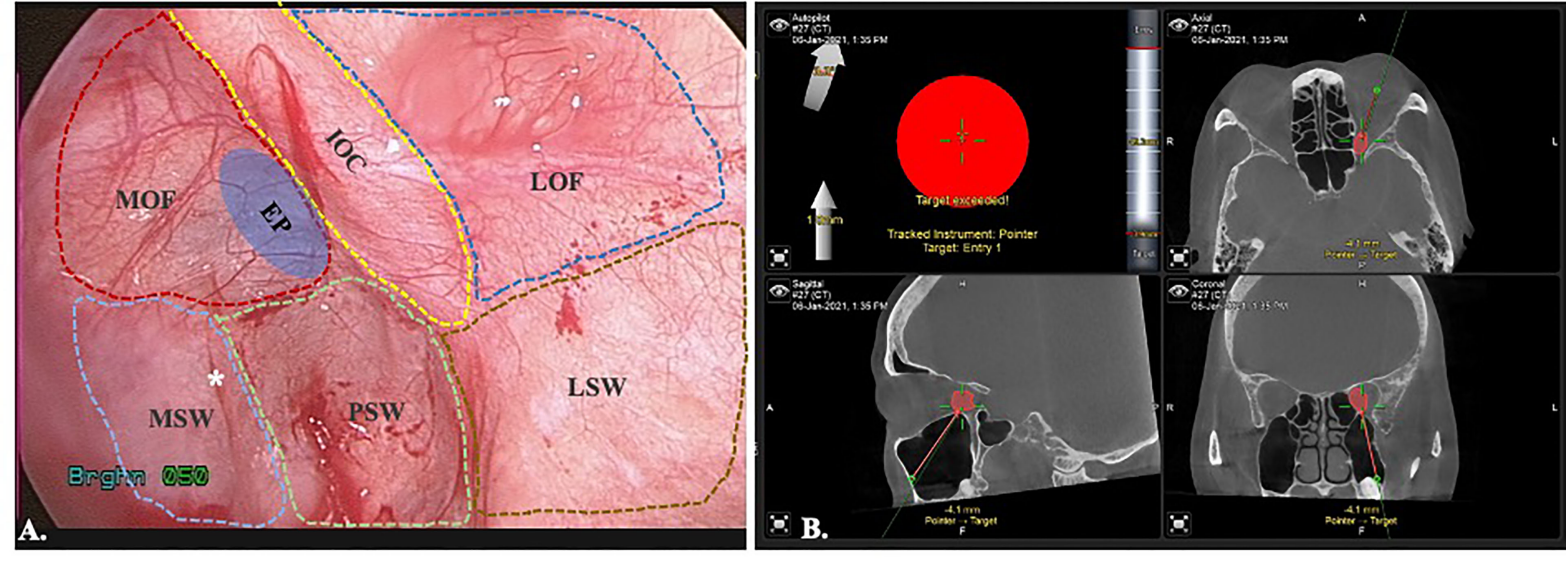
Endoscopic view of transmaxillary approach with the trajectorial-guided entry to the orbit. **(A)**. MOF, medial orbital floor; IOC, infraorbital canal; LOF, lateral orbital floor; MSW, medial sinus wall; PSW, posterior sinus wall; LSW, lateral sinus wall; EP, entry point; *maxillary ostium. **(B)** Trajectorial-guided orbital entry.

The surgeon position stands at the same site of the affected eye facilitate medial dissection of the orbit. The patient’s head position is upward extended and turned away from the surgeon side. Upper lip is retracted upward to reveal the buccogingival sulcus. Supravestibular incision to maintain a mucosal cuff is done from the canine to the second molar. The anterior wall of the maxillary sinus is exposed by elevating the subperiosteal flap. The infraorbital neurovascular bundle is identified and preserved. A rectangular bony window is prepared for antrostomy after pre-fixed a miniplate on the bone fragment to facilitate later reposition (**Figure 2**). To optimize the freedom of surgery, the bony window should be designed as large as possible for sufficient surgical corridor. The maxillary sinus is entered and a 0-degree, 4mm endoscope (Karl Storz and Co, Tuttlingen, Germany) is introduced to visualize the surgical field. There are two surgical landmarks identified: the infraorbital canal (IOC) and the maxillary ostium (**Figure 3A**). The IOC presents as linear protuberance running from the junction between the superior and posterior sinus walls to the infraorbital foramen. The IOC separate the orbital floor to a thinner medial part (1.0-2.0mm) and a thicker lateral part (2.5-4.0mm) (14). The maxillary ostium sited at the posterior-superior corner of the medial sinus wall. After identified the medial part of the orbital floor, the navigation pointer is introduced to locate the entry site. The reflective marker spheres can also attach onto the surgical instruments by adapter array for real-time guidance. The length, diameter, and vector of the instruments can be easily calibrated by the instrument calibration matrix. By combining the autopilot function, the trajectorial-guided surgical dissection could be done in accuracy and safe (**Figure 3B**). The periorbita is readily seen after the orbital floor is opened.

#### 2.2.3 Periorbita Dissection and Image-Controlled Tumor Excision

**Figure 4:**
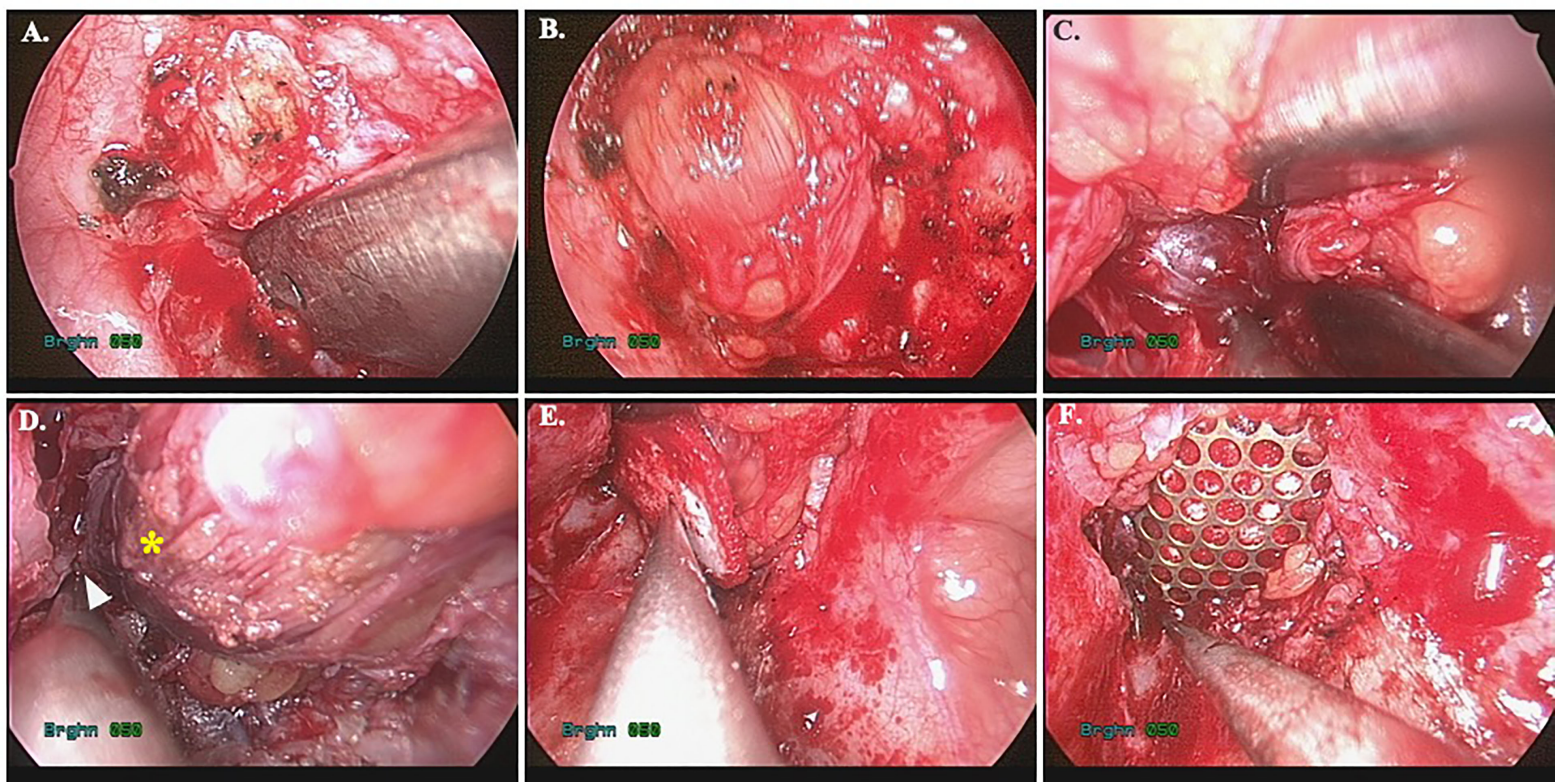
Image-controlled surgical excision of medial orbital tumor with reconstruction. **(A)** entry of the medial orbit; **(B)** periorbita and inferior rectus muscle; **(C)** identify the tumor; **(D)** image after tumor excision, the arrowhead: ethmoid sinus, star: medial rectus muscle; **(E)** bio-collagen membrane repair of the defect; **(F)** Ti-mesh reinforcement of the inferomedial strut.

After creation of the orbital floor window, the navigation pointer is used to locate the tumor position (**Figure 4A**). The surrounding bone is carefully removed with rongeur or rotating bur. It should be cautious that adequate protection is necessary to prevent periorbita from accidentally drawn in by the rotating airflow. This could result in catastrophic injury to the extraocular muscles and nerves. The incision is performed longitudinal to the long axis of the orbit. Once the periorbita is opened, the medial and inferior rectus muscles (MR and IR) are seen as dissection landmarks (**Figure 4B**). Extraconal lesions are usually easily identified and less need for dissection after periorbita incision and navigator identification (**Figures 4C, D**). For intraconal tumors, the MR and IR can mobilize medially/laterally and superiorly. For the lesions sited superomedially, a larger orbital floor surgical defect allows to mobilize the entire orbit inferiorly which provide access to the area (15, 16). With transmaxillary endoscopic approach, the surgical target could safely reach medially to the ethmoid sinus and even superiorly to the sphenoid sinus and optic canal under navigation control. After identifying the tumor, the dissection is proceeded under image controlled and the cryoprobe may assist in cryoextraction of the tumor in some scenario (17).

#### 2.2.4 Imaged-Controlled Medial/Inferior Orbital Walls Repair

**Figure 5:**
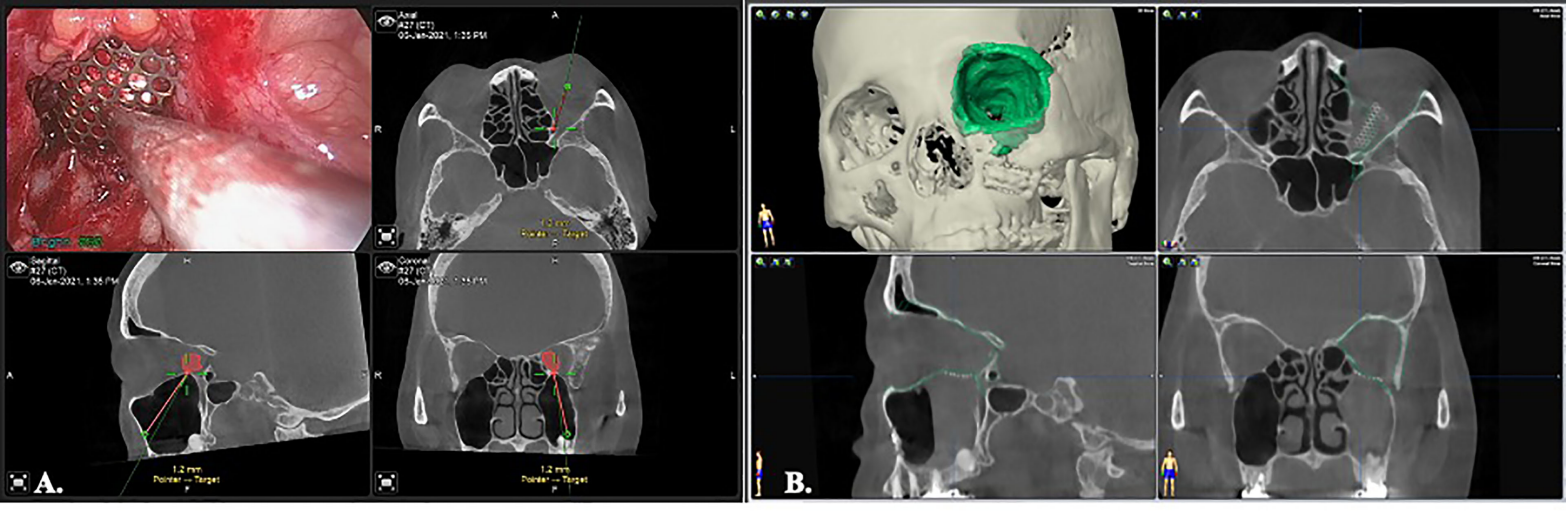
Image-controlled orbital floor reconstruction. **(A)** position control of the Ti-mesh by real time navigation. **(B)** post-op image showed high level of accuracy with orbital floor reconstruction. Green: pre-operative orbital wall.

The orbital floor (or with the medial wall defect) can be repaired straightforward after removal of the tumor. For small defects that are not involving the inferomedial strut, an alloplastic material, e.g. biocollagen membrane, or porous polyethylene implants is sufficient for endoscopic reconstruction (**Figure 4E**). For larger defects or those involving the inferomedial strut, the titanium mesh could be applied to reinforce the structure and prevent diplopia from orbital malposition (**Figure 4F**). The mesh can easily be pre-bended by the physical model and inserted into the surgical defect under endoscope. Navigation provides the accurate position control (**Figure 5**). Forced duction test should be performed to preclude any soft tissue entrapment by the material.

#### 2.2.5 Reposition the Anterior Maxillary Wall and Wound Closure

After completion of the orbital floor repair, the bony fragment of the maxillary anterior wall is repositioned and fixed with miniplate. The buccogingival incision is primarily close with resorbable sutures.

## Results

**Figure 6:**
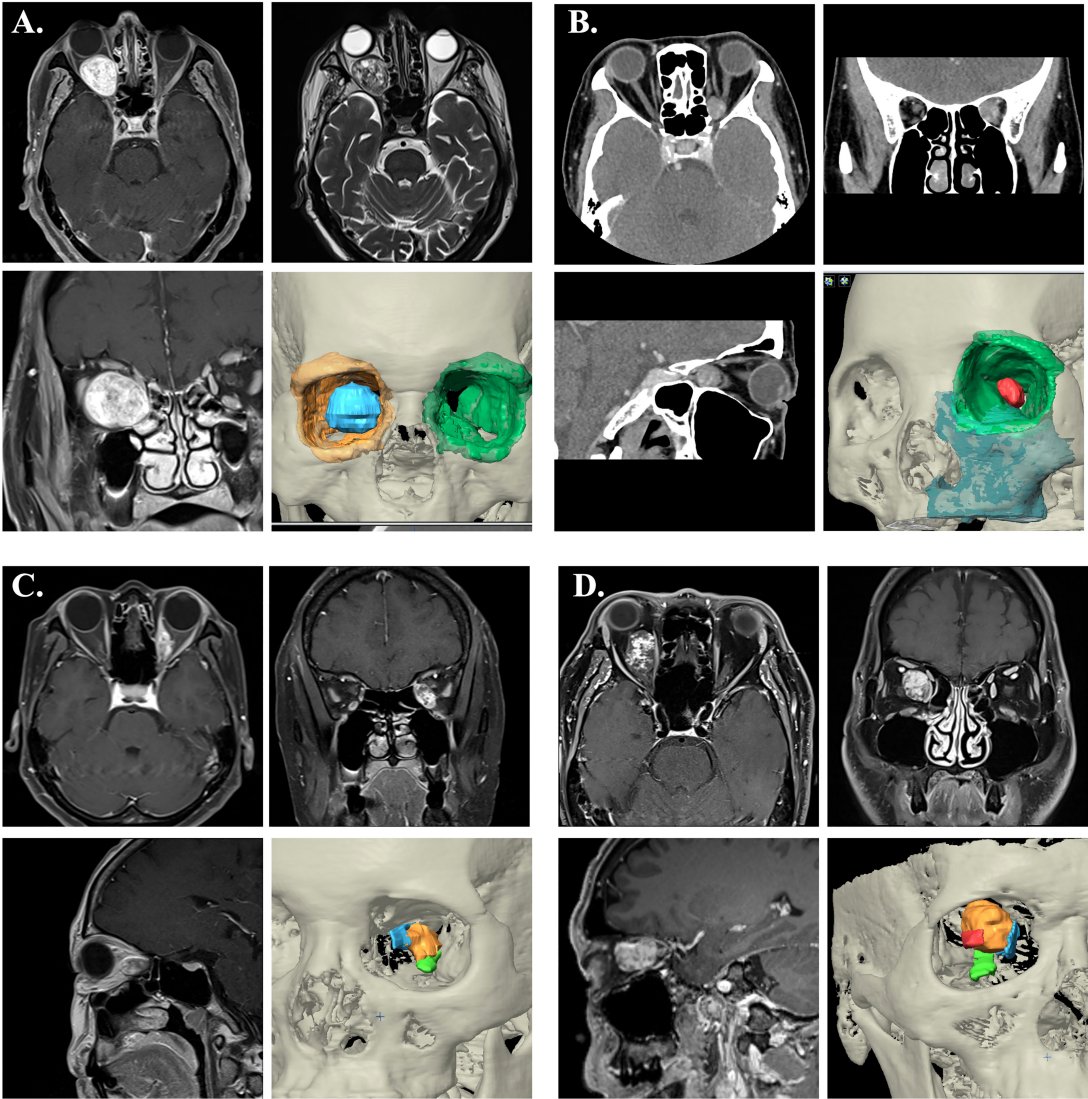
Image characteristics of the cases. **(A)** The contrast-enhanced MRI showed a retrobulbar intraconal well-demarcated ovoid mass of right orbit. The tumor showed heterogenous appearance on T2W sequences with some internal cystic component, and significant contrast enhancement. The tumor deviated the right optic nerve upward and causing right proptosis. 3D image rendering tumor lesion was demonstrated (blue). **(B)** The CT showed an ovoid, well-defined with contrast-enhanced mass at the medioposterior extraconal space and medial to the apical region of the optic nerve. 3D image rendering tumor lesion was demonstrated (red). **(C)** The MRI demonstrated an enhancing mass at medial side of left intraconal space (orange), which involving the medial rectus muscle (blue) and superior to inferior rectus muscle (green). **(D)** The MRI revealed a heterogeneously enhancing mass lesion at the right intraconal space (orange), medial to the optic nerve (red) that displaced laterally. (Blue: medial rectus muscle; Green: inferior rectus muscle)

Four cases with TMEA excision of medial orbital tumor were performed. All patients underwent surgery with radical intent, and a complete resection was obtained. The clinical characteristics were listed in the **Table 1**. Clinically, all patients had various degrees of visual impairment and proptosis. The individual patient presentation on the diagnosis were listed as followed:

Patient 1 was in a status of nearly complete vision loss due to optic nerve compression. Meanwhile, he had limited upward and lateral gaze of the affected orbit. The MRI showed a retrobulbar intraconal well-demarcated ovoid mass about 2.8x2.0x2.4cm. The tumor showed heterogenous appearance on T2W sequences with some internal cystic component, and significant contrast enhancement. The tumor deviated the right optic nerve upward and causing right proptosis (**Figure 6A**).

Patient 2 experienced mild symptoms with progressive blurred vision and proptosis. The CT showed an ovoid, well-defined with contrast-enhanced mass at the medioposterior extraconal space and medial to the apical region of the optic nerve. MRI images showed slight hypointensity in T1W sequences and hyperintensity in T2W sequences. The radiographic feature was favored of cavernous venous malformation (**Figure 6B**).

Patient 3 complained of painful swelling over left side orbit. The images showed a 1.2x0.9x2.1cm enhancing mass at medial side of left intraconal region, which involving the medial rectus muscle (**Figure 6C**). Suspected orbital lymphoma or idiopathic inflammation pseudotumor.

Patient 4 presented with progressive blurred vision for 2 years. The visual exam demonstrated decreased visual acuity, color sense, limited abduction, and mild exophthalmos of right sided eye. Orbital MRI revealed a heterogeneously enhancing mass lesion at the right intraconal space, medial to the optic nerve, and measuring about 2.0x2.3x1.7cm. With mass effect causing deviation of the right orbital nerve laterally. The lesion showed hyperintense T2 and hypointense T1FS appearance. Suspected cavernous venous malformation (**Figure 6D**).

**Figure 7:**
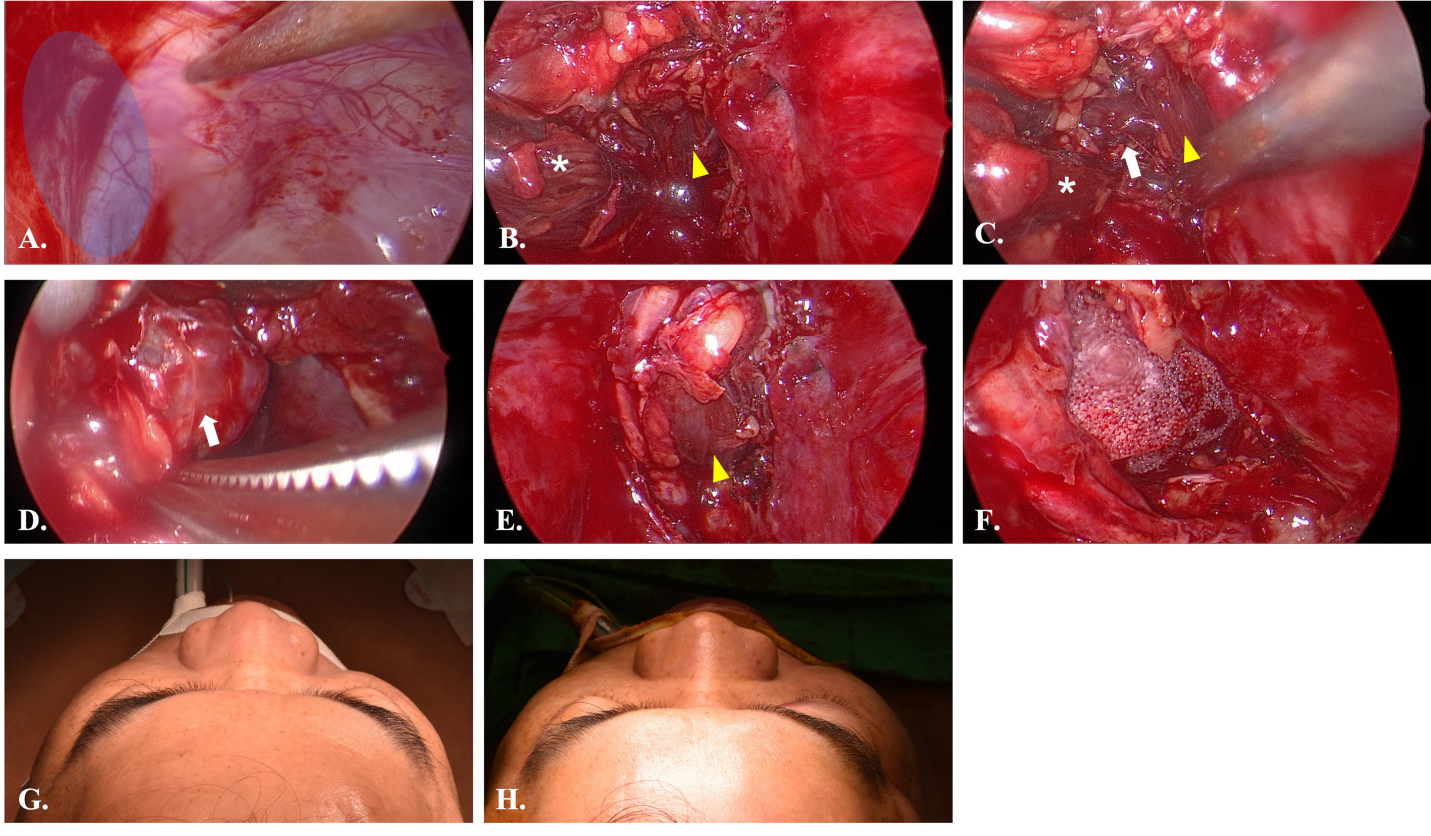
Intraoperative views of Case 4. **(A)** Endoscopic view of case 4. The pointer indicated the location of infraorbital nerve. The blue area is the entry point. **(B)** After incision of the periorbita, the inferior rectus muscle (yellow arrowhead) and medial rectus muscle (white star) were identified. **(C)** Dissection between inferior rectus and medial rectus muscle to identify the lesion (white arrow). **(D)** Dissection of the lesion from peripheral orbital tissue. **(E)** Return of inferior rectus muscle to original position after removal of the lesion. **(F)** The orbital floor was repaired with porous polyethylene mesh (MEDPOR). **(G)** Preoperative view showed mild proptosis of right eye. **(H)** Postoperative view showed symmetry orbital position.

**Figure 8:**
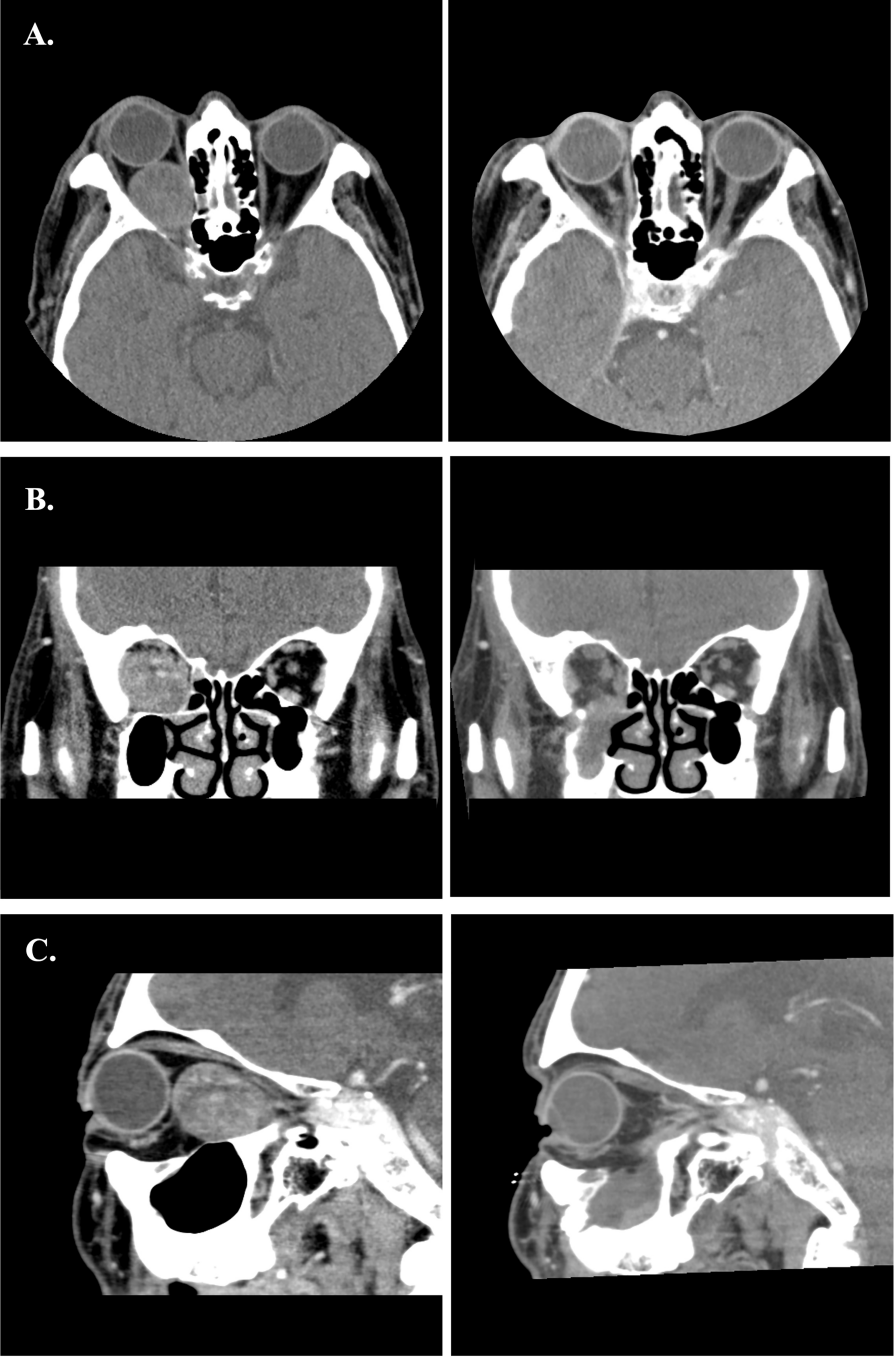
**(A–C)** Preoperative and postoperative images of Case 1. The left-side axial, coronal and sagittal images were the preoperative CT scans. The right-side postoperative CT images showed normal extraocular muscles position and well restored orbital volume.

**Figure 9:**
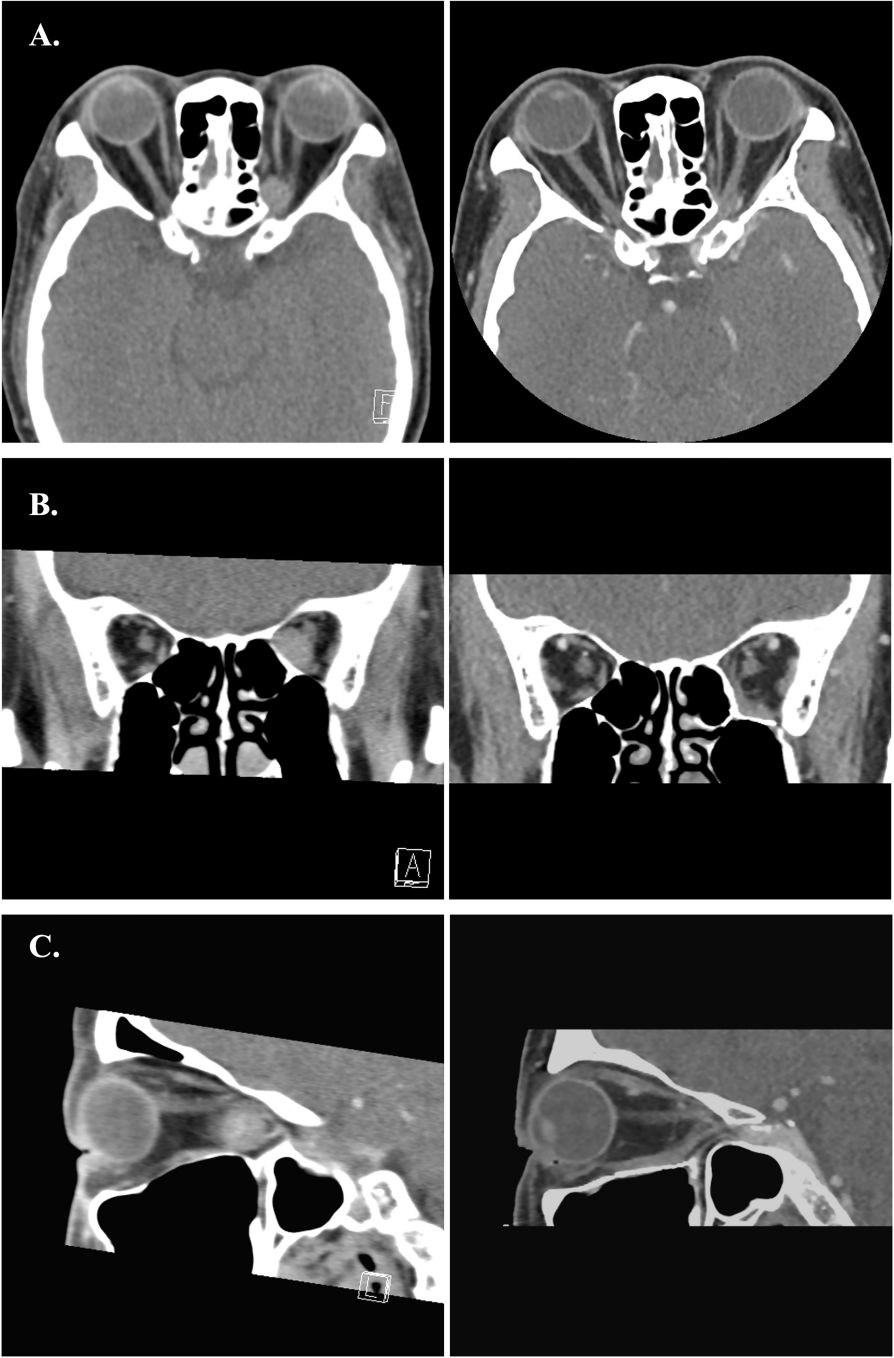
**(A–C)** Preoperative and postoperative images of Case 2. The left-side axial, coronal and sagittal images were the preoperative CT scans. The right-side postoperative CT images showed normal extraocular muscles position and well restored orbital volume with Titanium mesh.

**Figure 10:**
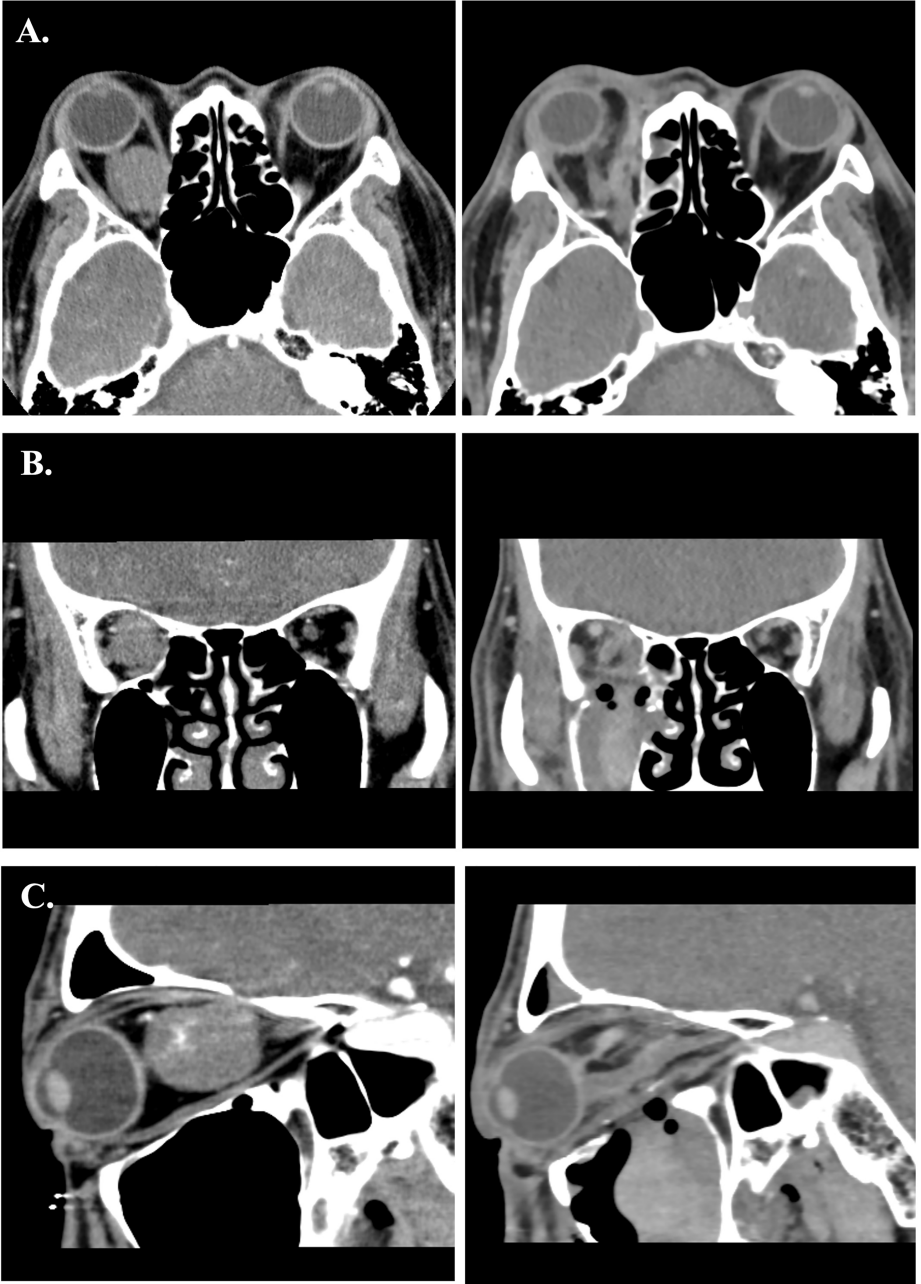
**(A–C)** Preoperative and postoperative images of Case 4. The left side axial, coronal and sagittal images were the preoperative CT scans. The right-side immediate postoperative CT images showed swelling of the medial rectus and mild distortion of medial rectus muscle.

**Figure 11:**
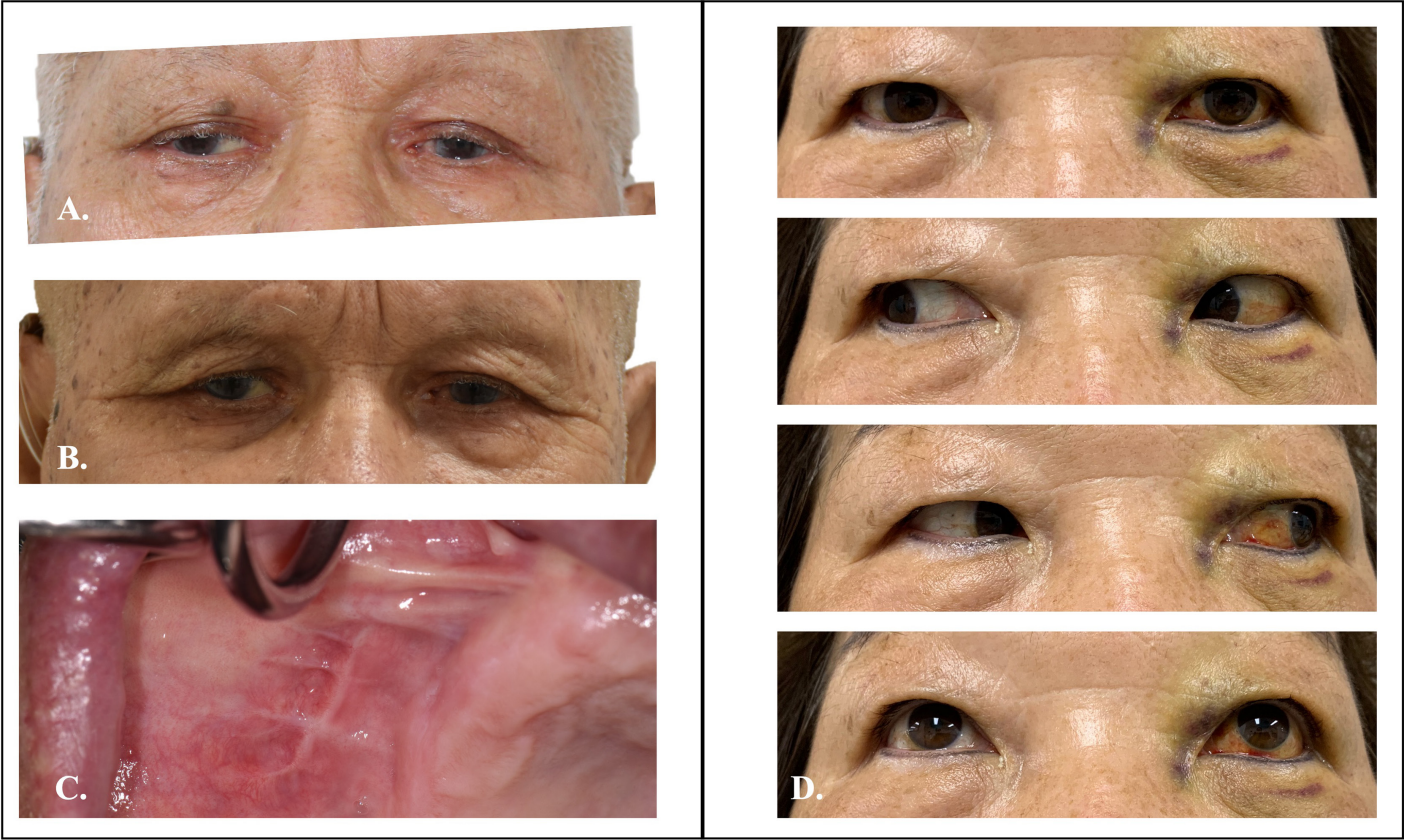
Preoperative and postoperative clinical photo of Case 1 and 2. **(A)** Preoperative photo of case 1 showed mild proptosis over right-side eye. **(B)** Symmetry of eye position after TMEA excision of orbital tumor. **(C)** Postoperative oral wound healed well. **(D)** Two weeks postoperative photo of case 2 showed well functional orbital movement.

Four lesions were difficult to approach by traditional transorbital route. We applied TMEA and successfully excised the lesions. For extraconal lesions, the surgical dissection was straightforward with limited periorbital dissection. However, the intraconal lesions need more muscle traction and may need fat extirpation especially close to the orbital apex. The surgical procedure in case 4 was shown in **Figures 7A–H**. The mean operation time were 202.3 ± 57.2minutes including the setup of navigator. The hospital stay was about 3.8 ± 0.8 days. All patients experienced a short duration of paranasal paresthesia, but all recovered within post-op 2 months except for the case 4. He still had numbness of the paranasal region at 2 months followed up. But the degree of numbness decreases over time. Patient 2 and 3 had post-op diplopia due to edematous change of the orbital content. It resolved within 2 months and no residual limitation of extraocular muscles was found. Patient 1 cannot evaluate the post-op diplopia due to near-complete vision loss before operation. However, his light perception immediate recover after operation. The visual acuity showed complete improved in patient 2 and dramatically improved in patient 1 from the initial no light perception state. The case 4 presented with persisted diplopia at upward and downward gaze after 2 months of followed up. However, the degree of diplopia showed progressive recovery. The summary of visual outcomes was listed in **Table 2**. Biocollagen membrane (ABCcolla^®^ Collagen Membrane, ACRO Biomedical Co., Ltd. Kaohsiung, Taiwan) was used for orbital floor reconstruction in case 1 and case 2 (**Figure 4E**). Titanium mesh (MatrixORBITAL ™ DePuySynthes, Oberdorf, Switzerland) was used for structural reinforcement of medial orbital struct in patient 2 (**Figure 4F**). Porous polyethylene implant (Medpor^®^, Stryker CMF, Kalamazoo, MI 49002, USA) was used in patient 4 (**Figure 7F**). Compared the reconstructive results, the patient 1 showed mild enophthalmos for only using biocollagen membrane reconstruction. In patient 2, 3 the post-operative CT images showed true-to-original reconstructed orbital floor without periorbita or muscular herniation (**Figure 9**). Preoperative and postoperative CT images of case 1,2,4 showed well restored orbital volume (**Figures 8**–**10**). The patient 4 has mild enophthalmos after operation even the CT images showed well bony reconstruction (**Figure 10**). The enophthalmos is suspected related to fat volume reduction during operation from its intraconal position and size. The clinical photo of case 1 and case 2 were shown in **Figure 11**. The images showed well orbital volume preservation after surgery and no interference on orbital movement.

**Table 2:** Preoperative and postoperative ophthalmic evaluation.

	Patient 1			Patient 2		
	Pre-op	Post-op 1wk	Post-op 2M	Pre-op	Post-op 1 wk	Post-op 2M
Visual acuity	NLP*	20/200	20/50	20/30	20/25	20/20
Hertel Exophthalmometer (mm)	14	9	9	16	Not perform	14
EOM	Restriction in all directions except abduction	No change	Mild to moderate improvement	Full and free	Mild restriction in supraaduction	Full and free
	**Patient 3**			**Patient 4**		
Visual acuity	20/20	20/20	20/20	20/40	20/20	20/20
Hertel Exophthalmometer (mm)	14/14	14/14	14/13	21	Not perform	16
EOM	Full and free	Mild infraduction and lateroduction limitation	Full and free	Full and free	Infraduction and supraduction	Progressive improve

*NLP, no light perception.

## 4. Discussion

Orbital tumor treatment is often challenging for both functional and esthetic considerations. Among them, orbital vascular malformations (OVMs) are the most common orbital masses, which are often misinterpretation as “tumor” in the context of cavernous hemangiomas, lymphangiomas … etc (18). In our cases, two were pathologically diagnosed as capillary venous malformation. In same study, Colletti et al. proposed three subtypes of OVMs, which are relevant to planning corresponding treatment modalities and predict the possible complications. Unlike the type 2 and type 3 OVMs which are connected to the venous system of the orbit (and sometimes intracranially), the type 1 OVM is excluded from the surrounding venous system. This feature allows relatively easiness on surgical dissection, and less bleeding during procedure. In terms of surgical approaches, transnasal endoscopic approach is fitted for the extraconal/intraconal type 1 OVM that situated at the medial quadrants of the orbit (18, 19). In this report, we proposed an alternative route to the medial OVMs from the floor. For the extraconal lesions, the dissection is relative straightforward. Once the lesion is localized under navigation, the periorbita was excised as needed and the dissection could be limited with endoscopic assistance. For the case 2 and 3, the patients experienced limited diplopia but fully recovered within 2 months is possibly related to post-operative tissue edematous change. For the intraconal lesions, dissection between inferior and medical rectus muscles is usually necessary. During procedure, the prolonged and excessive muscle traction will cause swelling and even ischemic change of the muscle that may cause diplopia after surgery (20–22). Especially when the lesion is close to the orbital apex, the surgical corridor is limited by the convergence of muscle cones which further increase the incidence of muscle injury. In our cases, although all the patients experienced diplopia immediate after operation. However, the diplopia is transient and all resolve in 2 months except the case 4. He still presented with limitation on upward and downward gaze after 2 months followed up but with progressive improvement. The position of this intraconal lesion was close to the orbital apex make surgical dissection more complicated. The postoperative images showed swelling of the inferior and medial rectus muscle reflect the intense inflammatory reaction and muscle dysfunction (**Figure 10**). This phenomenon is not seen in case 1. Although the lesion was also in intraconal position, it could be easily dissection off between medial and inferior rectus muscles from its more anterior location that made it away from the narrow apical region.

The traditional transorbital route has limited access to the posterior orbital tumors especially in the inferomedial and apical region (23). The operation on this tight, crowding space that packing with numerous critical neurovascular structures is just like “The devil’s touch”. Combination of extensive fronto-temporo-orbital-zygomatic dysjunction is sometimes indicated for complete excision, however, the potential neural complications, such as subdural hematoma, brain edema, CSF leakage, vision loss, postoperative seizure should be cautious (24) not mentioned to the complexity of the operation. Recently, the TNEA is gaining popularity for orbital tumor excision especially for intraconal lesions located inferiorly and medially to the optic nerve and the extraconal lesions adjacent to the paranasal sinuses (8, 19, 25, 26). Anatomically, the lamina papyracea is the only thin barrier between medial orbit and the ethmoid sinus. The transnasal route provides the shortcut to the orbit thus avoids plethoric muscle detachment and potential neurovascular compression from transorbital approach. The outcome of the purely TNEA excision of orbital tumors has been well elaborate in the systemic review (20). In this review, it demonstrated that TNEA can effectively excise a diverse array of intraconal/extraconal orbital lesions. However, almost one-third of cases had postoperative sequalae, which diplopia was the most frequent reported followed by enophthalmos. Although 76.2% of complications were transient, the overall complication rate was slightly higher than the reported rate from traditional orbitotomy procedures (21). Techniquewise, the TNEA usually begin with sphenoethmoidectomy to reach the lamina papyracea and then enter to the medial orbit after removing this thin bony barrier. The “sword fighting effect” that from narrow surgical corridor may limited the working channel for the intraconal lesions or those close to the orbital apex. To improve the maneuverability, the four-handed binostril procedure, extensive medial antrostomy, posterior septectomy may employ (27, 28). However, these maneuvers may increase the nasal complication rate and crusting (29). The TMEA provides an excellent operative window for instrumentation. According to the recent anatomical study from Donofrio CA et al. (30), the mean operative window for TMEA was 278.9 ± 43.8 mm^2^, which was significantly larger than the TNEA (240.8 ± 21.5mm^2^). The larger operative window improves the maneuverability of instruments. Furthermore, the surgical path of TMEA is a direct line of sight which is more intuitive for surgeons. The ample space from maxillary sinus tolerates different surgical instruments in the same channel. By creating a large antrum window, we could deploy the endoscope and dissecting instruments in a direct and single surgical port. The dissection was done straightforward, and the operation time could be controlled within 3 hours including the setup of navigation and orbital wall reconstruction. The distance of pivotal point of the instruments to the orbital floor is greater in TMEA, which produces a larger angle of attack than TNEA (45°-65° vs. 20°-30°, respectively) (30). Meaning that TMEA could cover the entire orbital floor as surgical field. In our experience, the TMEA could easily target posteriorly to the medial orbital apex and superiorly to the lateral wall of the sphenoid sinus after removing the orbital floor. According to the comparative study of different endoscopic approach to the anterolateral skull base, TMEA offers better surgical freedom and head-on approach than TNEA (31). This superior surgical flexibility is well demonstrated in our case 2. The lesion was located at the inferomedial aspect of the orbital apex and lateral to the sphenoid sinus. With the three-dimensional image rendering, virtual surgical trajectory was planned preoperatively. The orbital floor osteotomy and surgical dissection was accurately executed by real-time navigation control. The surgical freedom of TMEA is primarily limited by the size of antrum window. For patients with atrophic maxillary sinus, with mixed dentition stage, incomplete pneumatization are not indicated for this approach (32). We recommend creating the antrum window as large as possible, even including the entire anterior maxillary wall but the infraorbital foramen to avoid nerve injury. By using piezo osteotomy, the bone cutting is precise and atraumatic which facilitate later reposition of the bone fragment with miniplates. This temporary antrum window protocol preserves the integrity of the maxillary sinus, which reduces the soft tissue contracture on the surgical site and formation of oro-antral fistula. Although transient facial numbness may develop from traction injury to the infraorbital nerve, all our patients completely recovered within 2 months. The reported rhinological morbidities in TNEA such as epistaxis, nasal synechiae, nasal crusting, nasolacrimal duct injuries are free from TMEA (28, 33).

The other advantage from the ample surgical field provided by TMEA is the possibility of reconstruction of the orbital wall. The orbital floor osteotomy created for surgical access could be repaired under endoscope by alloplastic materials, such as collagen membrane, porous polyethylene implants, or titanium plates depending on the defect size and the strength need. The implant is easily inserted through the antrum window and placed between the periorbita and the bony orbit. Navigation is then used to ensure the position is true to original. This procedure prevents the herniation of the periorbita into the sinus, which avoid post-operative enophthalmos and long-term diplopia. In the literature, the risk of developing post-operative enophthalmos is 5.9% for TNEA, which is mainly due to fail to restore the orbital volume (34). Currently, there is no consensus on the best reconstruction option for TNEA, although the bone fragments, pedicle naso-septal flap, or allograft had been used (35). The limited surgical corridor in TNEA makes the medial or inferior orbital walls reconstruction difficult and most of the surgeons choose not to repair it. However, there were several excellent results of medical wall reconstruction by porous polyethylene mesh after traumatic injury and tumor excision from TNEA by Colletti et al. (36–40). With experienced hands, the medial wall defects could be repaired *via* TNEA and further prevent post-operative enophthalmos. The TMEA creates defects at the orbital floor, either biocollagen membrane or porous polyethylene mesh is suitable material for reconstruction. However, if the defects across the orbital supporting construct, more rigid material, e.g. Titanium mesh is the material of choice. With the experience on endoscopic orbital floor reconstruction in blow out fracture patients, orbital wall repair with Titanium mesh *via* TMEA is feasible and has more controllable outcome with the assistance of endoscope and navigation (41, 42).

## 5. Conclusion

Endoscopic surgery opens an eye for minimal invasive orbital tumor excision. The TMEA provides a versatile surgical corridor to the inferior and medial orbit and even to the orbital apex region. It provides an intuitive dissection corridor and an alternative surgical view to TNEA. In this computer-assisted workflow, we implement virtual surgical planning, intraoperative navigation, and true-to-original orbital wall reconstruction that is beneficial for patients by shorten surgical time, hospital stays, and minimal complications. We believe the application could be expanded to most of the orbital tumor surgery.

## Data Availability Statement

The original contributions presented in the study are included in the article/supplementary material. Further inquiries can be directed to the corresponding author.

## Ethics Statement

The studies involving human participants were reviewed and approved by Taipei Veterans General Hospital. The patients/participants provided their written informed consent to participate in this study. Written informed consent was obtained from the patients for the publication of any potentially identifiable images or data included in this article.

## Author Contributions

C-HW: protocol design, manuscript preparation, data collection, analysis, and final approval of the manuscript. Y-YH, T-LL, T-YW, H-CC, and C-CT: data collection and analysis. T-YW: image processing and virtual surgical planning. All authors are responsible for the manuscript. All authors contributed to the article and approved the submitted version.

## Funding

This work was funded by V110A-006 from Taipei Veterans General Hospital research grant.

## Conflict of Interest

The authors declare that the research was conducted in the absence of any commercial or financial relationships that could be construed as a potential conflict of interest.

## Publisher’s Note

All claims expressed in this article are solely those of the authors and do not necessarily represent those of their affiliated organizations, or those of the publisher, the editors and the reviewers. Any product that may be evaluated in this article, or claim that may be made by its manufacturer, is not guaranteed or endorsed by the publisher.
